# Creation of GMP-Compliant iPSCs From Banked Umbilical Cord Blood

**DOI:** 10.3389/fcell.2022.835321

**Published:** 2022-03-16

**Authors:** Pei Tian, Andrew Elefanty, Edouard G. Stanley, Jennifer C. Durnall, Lachlan H. Thompson, Ngaire J. Elwood

**Affiliations:** ^1^ Blood Development, Murdoch Children’s Research Institute, Melbourne, VIC, Australia; ^2^ Department of Paediatrics, The University of Melbourne, Melbourne, VIC, Australia; ^3^ Immune Development, Murdoch Children’s Research Institute, Melbourne, VIC, Australia; ^4^ Florey Institute of Neuroscience and Mental Health, Melbourne, VIC, Australia; ^5^ BMDI Cord Blood Bank, Melbourne, VIC, Australia

**Keywords:** induced pluripotent stem cells, IPSC, reprogramming, GMPcompliant, xeno-free, cord blood, clinical -grade, cord blood bank

## Abstract

Many clinical trials are in progress using cells derived from induced pluripotent stem cells (iPSC) for immunotherapies and regenerative medicine. The success of these new therapies is underpinned by the quality of the cell population used to create the iPSC lines, along with the creation of iPSCs in a fully Good Manufacturing Practice (GMP)-compliant environment such that they can be used safely and effectively in the clinical setting. Umbilical cord blood (CB) from public cord blood banks is an excellent source of starting material for creation of iPSCs. All CB units are manufactured under GMP-conditions, have been screened for infectious diseases, with known family and medical history of the donor. Furthermore, the HLA tissue typing is known, thereby allowing identification of CB units with homozygous HLA haplotypes. CB cells are naïve with less exposure to environmental insults and iPSC can be generated with high efficiency. We describe a protocol that can be adopted by those seeking to create clinical-grade iPSC from banked CB. This protocol uses a small volume of thawed CB buffy to first undergo *ex-vivo* expansion towards erythroid progenitor cells, which are then used for reprogramming using the CytoTune™-iPS 2.0 Sendai Reprogramming Kit. Resultant iPSC lines are tested to confirm pluripotency, genomic integrity, and stability. Cells are maintained in a feeder-free, xeno-free environment, using fully defined, commercially available reagents. Adoption of this protocol, with heed given to tips provided, allows efficient and robust creation of clinical-grade iPSC cell lines from small volumes of cryopreserved CB.

## 1 Introduction

The potential clinical utility of cells derived from induced pluripotent stem cells (iPSC) is unlimited. The first published clinical trial examined the safety and efficacy of iPSC-derived mesenchymal stromal cells in acute steroid-resistant graft versus host disease (Bloor et al., 2020). There are now many pre-clinical and clinical trials in progress using iPSC-derived cells for cellular therapies (Kim et al., 2022), ranging from regenerative repair for cardiac and neural disease through to immunotherapies for the treatment of cancer (Cyranoski, 2018; Stoddard-Bennett and Reijo Pera, 2019; Sugai et al., 2021).

Umbilical cord blood (CB) stored in public cord blood banks (CBB) is an excellent source of starting material for the creation of iPSCs (Zhou and Rao, 2015). Unlike skin fibroblasts (Abyzov et al., 2012), CB cells are minimally exposed to environmental factors. Moreover, each CB unit stored in a public CBB is accompanied by a full family medical history, follow-up data and results from screening for infectious diseases. All CB units listed for search on the international bone marrow donor registries will have been manufactured in a facility under Good Manufacturing Practice (GMP), with most countries requiring oversight by their Regulatory Authorities. Many public CBBs also hold international accreditation through the Foundation for the Accreditation of Cellular Therapy (FACT), providing assurance of compliance with worlds’ best practice. Furthermore, all CB in public CBBs will have known Human Leukocyte Antigen (HLA) genetic sequence, thereby readily allowing the identification of CB units with homozygous HLA haplotypes that may act as “Super Donors” (Taylor et al., 2012; Sullivan et al., 2020). The BMDI CBB, a public CBB, was established in 1996, holds a GMP manufacturing licence from the Therapeutic Goods Administration (TGA) and is internationally accredited by FACT. Each CB unit is cryopreserved and stored with contiguous segments and pilot vials of representative viable cells; these fractions can potentially be used for reprogramming without compromising the banked CB units which were collected for the purpose of bone marrow transplantation. The key challenge in using this small fraction is that the amount of CB available for reprogramming is limited. Therefore, an optimised protocol with high cell recovery and reprograming efficiency is necessary to generate enough iPSC clones.

There are at least three considerations in the production of cGMP-compliant iPSC lines. The first is to ensure that all activities are undertaken within a robust Quality Systems framework. Linking into the Quality Systems already in place at the BMDI CBB will ensure that the iPSC lines created will ultimately meet GMP compliance with respect to documentation and operations. The second consideration is the establishment and use of appropriate Quality Assurance (QA) and Quality Control (QC) parameters and assays to ensure both efficacy of the cells produced, and safety of the cells to be used within a clinical setting. To this end, the collaborative efforts by the Global Alliance for iPSC Therapies (GAiT) have defined the minimum QA and QC criteria as best current practice (Sullivan et al., 2018). The third aspect to producing clinical grade iPSC lines relates to the starting cell material and the critical materials used to derive the lines. It is this aspect we focus on in this paper (Supplementary Figure S1), to establish a pipeline of iPSC generation with suitable starting material and commercially available reagents which are manufactured in cGMP compliant facilities. The estimated timeline and cost of these technical considerations, without the facility, labour and quality management costs, can be found in the Supplementary Figure S2.

Our long-term goal is to create a bank of iPSC cell lines from CB with common homozygous haplotypes, that will be suitable for downstream clinical application. Here we establish a protocol to generate clinical-grade iPSC cell lines from a limited amount of cryopreserved CB as the starting material, using commercially available media and reagents. We believe that this protocol could be adopted seamlessly in any cGMP compliant facility to generate clinical-grade iPSC cell lines.

## 2 Materials and Methods

### 2.1 Starting Material

#### 2.1.1 Cryopreserved CB

CB samples were obtained from the BMDI CBB with approval of the Royal Children’s Hospital Melbourne Human Research Ethics Committee (HREC 36127A) and with consent for research from CB donors.

For this study, only CB units that did not meet CBB criteria for release for bone marrow transplant were used. The CB units were processed and cryopreserved using standard CBB procedures. Aliquots of CB buffy coat (0.5 ml) were stored in liquid nitrogen vapour phase at −196°C prior to thaw and erythroid cell expansion.

### 2.1.2 Erythroid Cell Expansion and Characterization

The cryopreserved CB aliquots were thawed in a 37°C water bath, DMSO was removed by gently mixing the thawed blood with 4 volumes of StemSpan™-ACF (StemCell Technologies 09805), then centrifuged at 300 x g for 10 min.

**FIGURE 1:**
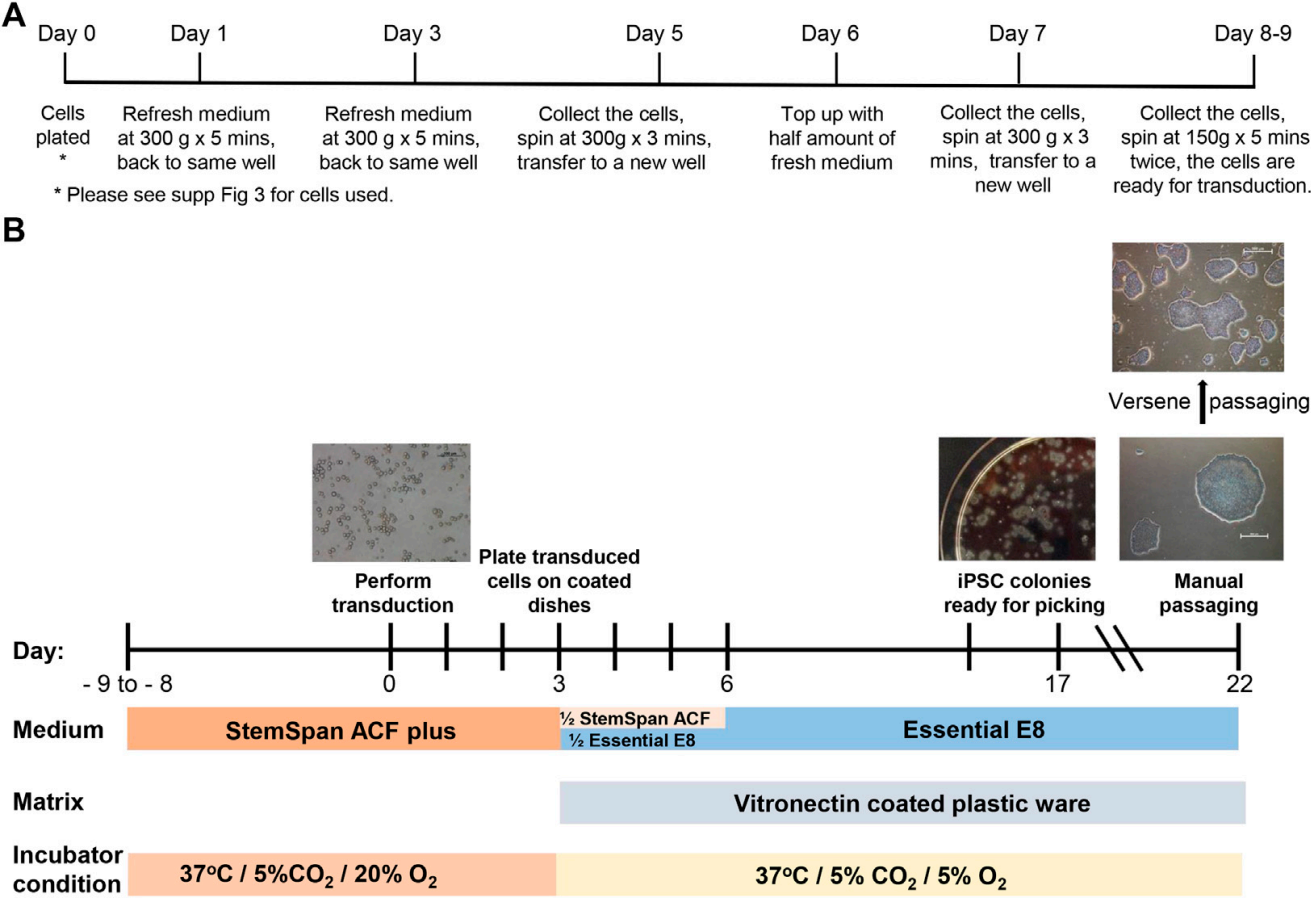
Erythroid cell expansion and reprogramming timeline. **(A)** The timeline and details of the media refreshing procedure during cell expansion. **(B)** Major steps of cord blood reprogramming procedure.

A protocol developed by Vlahos et al. (Vlahos et al., 2019) and used by the iPSC Core Facility at the Murdoch Children’s Research Institute (MCRI) was modified to be compliant with GMP-like cell culture conditions. For erythroid cell expansion, the thawed CB blood cells (Supplementary Figure S3) were resuspended in 1 ml StemSpan™-ACF containing 1x StemSpan™ Erythroid Expansion Supplement (100x) (StemCell Technologies 02692) (StemSpan-ACF plus), transferred into the well of a 12-well plate (ThermoFisher 150628) and incubated at 37°C with 5% CO_2_ and 20% O_2_ overnight. On the following day and every other second day (not more than 9 days in total), the media was refreshed with freshly prepared StemSpan-ACF plus (Figure 1A).

Cells grown in culture were characterised by Flow Cytometry. The cells were briefly centrifuged at 350 x g for 5 min and resuspended in 50 µL of antibodies cocktail containing: PE-Cy7 conjugated CD235a/GlyA (1:100; Beckman Coulter A71564), FITC conjugated CD3 (1:25; Beckman Coulter IM1281U), APC conjugated CD71 (1:100; Biolegend 334108) and BV-421 conjugated CD19 (1:50; BioLegend 302234) at 4°C for 15 min in the dark. The stained cells were washed twice with PBS +2% FCS and centrifuged at 350 x g for 5 min. The samples were run on a BD LSRFortessa™ cell analyser and the results were analysed using FCS Express.

### 2.2 Reprogramming

#### 2.2.1 Erythroid Cell Reprogramming

The CytoTune™-iPS 2.0 Sendai Reprogramming (Figure 1B) Kit (Invitrogen A16517) was used according to manufacturer’s recommendations with minor modifications. The transduction was performed at a multiplicity of infection (MOI) of 5 in StemSpan-ACF plus supplement containing 4 μg/ml of Polybrene (Sigma-Aldrich TR-1003) in a 12-well plate by centrifugation at 2000 RPM for 30 min at RT, then the cells were incubated at 37°C, 5% CO_2_ and 20% O_2_ or 5% O_2_ overnight. Exactly 24 hours after transfection, Sendai Virus was removed by centrifugation and the cells resuspended in 2 ml freshly prepared StemSpan-ACF plus. The cells were then incubated at 37°C, 5% CO_2_ and 20% O_2_ or 5% O_2_ for 2 days (as described in Results).

For *in vitro* culturing, the plastic plates and flasks were pre-coated with Vitronectin XF™ (StemCell Technologies 07180) or Vitronectin (VTN-N) (ThermoFisher A31804) (0.5–1 μg/cm^2^) or rhLaminin-521 (ThermoFisher A29249) according to manufacturer’s instructions. On day 3, the transduced cells were counted and resuspended in freshly prepared StemSpan-ACF (no addition of supplement) mixed with Essential 8 (Gibco A1517001) or Essential 8 Flex (Gibco A2858501) at 1:1 ratio. Transduced cells were plated at 30,000 and 50,000 per 4 ml on pre-coated 6 cm dishes. The next day, 2 ml of Essential 8 medium or Essential 8 Flex medium was added into each dish. On day 6, media was completely refreshed and changed daily until days 19–21. Each individual colony with iPSC-like morphology (round clear tight edges and uniform surface) was then transferred from the dish to an individual well of a 12-well plate (P0) by manual passage. For each individual colony, manual passage was performed in the following way, using the microscope housed within the BSC with eyepiece protruding through the front glass panel. Each colony was dissected using sterile needles into 6–12 pieces (depending on size of the colony). All pieces were then transferred into one well of a 12-well plate. After 3–4 days with refreshing the media daily, the 6–12 pieces had become 6–12 colonies within the well. Only one or two of these 6–12 colonies were transferred, both into the same well of a 6-well plate (ThermoFisher 140675). The one or two colonies selected were those that displayed the best iPSC-like morphology, the remaining colonies were discarded. This manual passaging was repeated 4 times. For one reprogramming, usually 10–15 colonies (presumed to be clones) were chosen to go through this manual passaging. During this process, any colonies that continually displayed loose cells and an uneven surface were discontinued. Following the series of manual passages, cells were then passaged from a 6-well plate to a T25 flask (ThermoFisher 156367) by an EDTA-based (Versene, Gibco 15040066) “dry” passaging method (After adding Versene, cells were incubated at 37°C for 1 minute, Versene was removed and cells incubated for a further 2 min 37°C, cells were then collected using growth medium). After 3-4 passages in T25 flask (at 1:3 to 1:6 dilutions), three “clones” with the best morphology (i.e. tight cell mass and clear edge) were expanded further for analysis and cryopreserved in PSC Cryomedium according to manufacturer’s instructions (Gibco A2644401). The remaining “clones” were cryopreserved as backup clones. During manual passaging and the EDTA-based passaging steps, all the media and buffers were brought to RT prior to use. When Vitronectin coated plasticware was used, RevitaCell™ Supplement (Gibco A2644501) was added at 1:200 dilution in E8 medium at passaging and removed after 3–18 h incubation.

### 2.3 iPSC Characterisation

#### 2.3.1 Flow Cytometry Analysis

Cultured cells at P4+4 stage (4 manual single colony passages plus 4 EDTA-based passaging on T25 flask) were dissociated with 1 x TrypLE (Gibco 12563029) into single cells. For panel 1 analysis (TRA-1-81 and OCT3/4), 5 × 10^5^ single cells were fixed with BD Cytofix™ Fixation Buffer (BD 554655) for 15 min at 4°C then permeabilized with 1x BD Perm/Wash™ Buffer (BD 554723) for 20 min at 4°C. The permeabilized cells were stained with 50 µL of antibodies cocktail (diluted in 1x BD Perm/Wash™ Buffer) for 40 min at 4°C in dark and washed twice with 1x BD Perm/Wash™ Buffer. For panel 2 analysis (TRA-1-81, TRA-1-60, SSEA4, SSEA1), 5 × 10^4^ to 1 × 10^5^ single cells were stained with 25 µL of antibodies cocktail for 15 min at 4°C in the dark and washed twice with PBS +2% FCS. The cells were then resuspended in 1 x PBS and run on a BD LSRFortessa™ cell analyser. The result was analysed using FCS Express. The following antibodies were used: AF647 conjugated TRA-1-81 (1:50; BioLegend 330706), PE conjugated OCT3/4 (1:25; BD 560186), PE conjugated TRA-1-60 (1:50; BioLegend 330610), A488 conjugated SSEA4 (1:25; BioLegend 330412), BV-421 conjugated SSEA1 (1:25; BioLegend 125614).

#### 2.3.2 *In vitro* Directed Differentiation Assays

To examine the pluripotency of the putative iPSC lines, the reprogrammed cells at P4+4 stage were differentiated towards three germ layers: neural stem cell (NSC) (ectoderm), cardiomyocytes (mesoderm) and definitive endoderm. *In vitro* differentiation of the iPSC lines utilised published methods which have been referenced and described in detail within the Supplementary Data.

### 2.4 Genomic Integrity

One T25 flask of cells at 70–80% confluence was dissociated with Versene and washed with 1 x PBS once. The cell pellet was sent to the Victorian Clinical Genetic Services (VCGS) for SNP array analysis using Illumina Infinium CoreExome-24 v1.1 array. The result was analysed using hg19/GRCh37 (February 2009) assembly with 0.5 Mb resolution.

### 2.5 Mycoplasma Testing

Mycoplasma testing was performed by a commercial service provider, Cerberus Sciences (Adelaide, Australia) with ISO 9001 certification who performed a PCR-based test to detect *M. arginini, M. hominis, M. hyorhinis, M. fermentans, M. orale, M gallisepticum, Acholeplasma laidawii*.

### 2.6 Reagents

Each reagent used in this study was carefully selected, commercially available and manufactured in a cGMP facility or chemically defined, thereby assuring their quality and consistency (Table 1). Potential variation caused by different batch number and lots of reagents was controlled for by ensuring validation experiments performed using the same iPSC line (for example comparing normoxic and hypoxic conditions) used the same batch and lot number of reagents.

**TABLE 1:** The composition of chemically defined and xeno-free materials for hiPSC generation. Materials include the starting material, reprogramming kit, buffers and solutions used in the iPSC generation. Xeno-free: non-human-derived components; ACF: animal component-free; TGA: Therapeutic Goods Administration; FACT: Foundation for the Accreditation of Cellular Therapy; CTS: Cell Therapy Systems https://www.thermofisher.com/au/en/home/clinical/cell-gene-therapy/cell-therapy/cell-therapy-systems.html?SID=fr-ctsystems-main. Products in the CTS column allow researchers to move their cell products from bench to clinical trial.

Procedure	Starting material/Medium/kits/Solution	Xeno-free	ACF	cGMP manufactured	TGA/FACT	CTS
Starting material	Banked CB	√			√	
Erythroid expansion (STEMCELL Technologies)	StemSpan™-AOF (# 09860)		√			
	StemSpan™ Erythroid Expansion Supplement (100X) (# 02692)	√				
Reprogramming (Gibco™)	CytoTune^TM^-iPS 2.0 Sendai Reprogramming Kit (A34546)					√
	Essential 8™ Medium (A2656101)	√		√		√
	Vitronectin (VTN-N) (A27940)		√	√		√
iPSC Expansion (Gibco™)	Versene Solution (A4239101)		√	√		√
	RevitaCell™ Supplement (A4238401)		√	√		√
	DPBS (1X), (Ca^−^/Mg^−^) (A1285601)		√	√		√
	Vitronectin (VTN-N) (A27940)		√	√		√
iPSC Cryopreservation	PSC Cryomedium (A4238801)	√		√		√

## 3 Results

### 3.1 Erythroid Cell Expansion

Based on the protocol used in the iPSC core facility at MCRI, an expanded erythroid progenitor cell population was used for reprogramming. To obtain enough cells for reprogramming and to avoid using Ficoll (Fuss et al., 2009), a protocol was optimised that included a thaw-washing step to isolate mononuclear cells (MNC) for the establishment of *ex-vivo* cultures. Optimisation of the thaw-washing step showed that the most effective erythroid cell expansion was achieved by slowly adding 4 x volume of StemSpan™ to the thawed CB buffy, mixing the cells gently then centrifugation for 10 min at 300 x g (data not shown). Following centrifugation, three layers were observed; a reddish cell pellet on the bottom, a non-transparent dark red layer in the middle and a transparent light red media layer on the top (Supplementary Figure S3). Following removal of the top light red layer, best erythroid cell expansion results were achieved by taking a mix of the cell pellet and middle layer together for establishment of *ex-vivo* cultures (data not shown).

**FIGURE 2:**
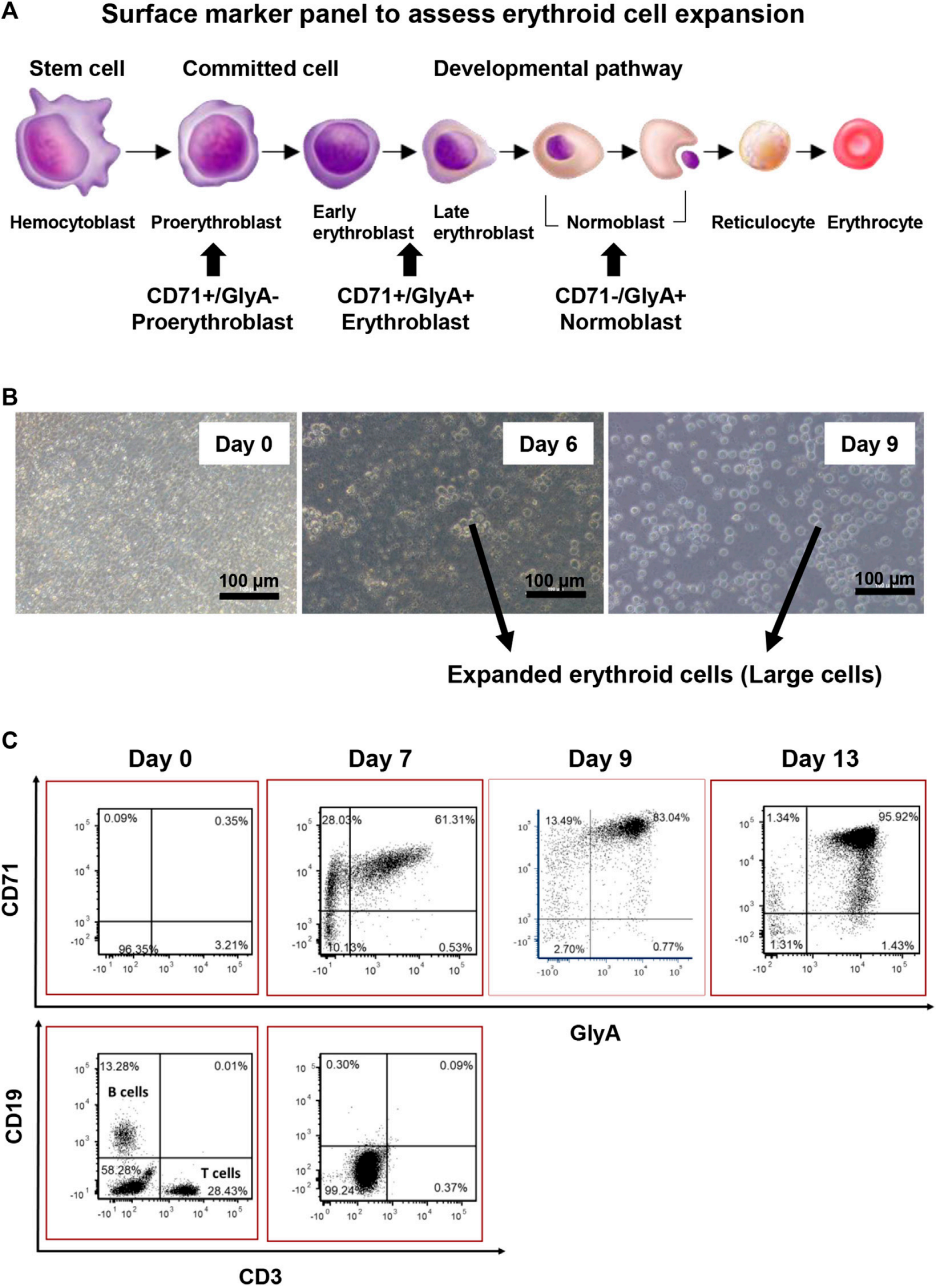
Characterisation of erythroid cell expansion. **(A)** Panel of Surface markers chosen for assessing erythroid cell expansion. **(B)** Expanded erythroid cells are larger in size compared with the input MNCs. **(C)** 2D plot of erythroid cell expansion. The erythroid cell cultures should not exceed 9 days as cells proceed to become normoblasts.

A panel of surface markers was established to monitor the erythroid cell expansion progress (Figure 2A). The desired erythroid cell progenitor phenotype for reprogramming is that of an erythroblast with the cell surface phenotype of CD71^+^/GlyA^+/−^, and which was confirmed to be the phenotype of the “large” cells (compared to starting cells), around 10.9–14.9 µm in diameter, that first appeared on day 4 in culture (Figure 2B) and were shown by flow cytometry on day 7 to be CD3 and CD19 negative (Figure 2C). Only these large cells were counted to assess the erythroid cell expansion efficiency.

The longer the *ex-vivo* culture time, the higher the erythroid cell yield. However, to prevent over culturing the cells, the surface marker expression patterns of the cultured cells were investigated on day 7, 9, and 13. On day 7, the percentage of erythroblasts (CD71^+^, GlyA^+/−^) increased from 5 to 90%, and the percentage of B cells and T cells dropped to below to 1%. From day 7 to day 13, the total percentage of CD71^+^ cells (GlyA^+/−^) remained the same. From day 7 to day 9, a significant shift of CD71^+^/GlyA^−^ cells to CD71^+^/GlyA^+^ was observed. On day 13, a high number of GlyA^+^/CD71^dim^ cells appeared, with some cells having become GlyA^+^/CD71^-^ (normoblasts) (Figure 2C). Therefore, the longest period of *ex-vivo* cell expansion should not exceed 9 days prior to reprogramming.

A “clean-up” step (to reduce the amount of debris and other types of cells) was employed prior to the cells being used for reprogramming. Because the size of the desired erythroid cells (erythroblasts) was much larger than the other cells and debris in the medium, an optimised centrifugation step was introduced. Centrifugation at 150 x g for 5 min gave the best “clean-up” without compromising the erythroid cell number (data not shown).

### 3.2 Erythroid Cell Reprogramming

The reliability and reproducibility of Sendai virus reprogramming of CB erythroid cells was assessed using two different CB units (#23 and #26), along with technical replicates for CB unit #26. Transduction efficiency (n = 4) was assessed by counting the total number of iPSC-like colonies divided by the number of erythroid cells used for reprogramming and was 0.03–0.19%.

Transduction efficiency was around 1.5-fold greater, with colonies larger in size, when reprogrammed cells were maintained at 5% O_2_ compared to 20% O_2_ (Supplementary Figure S4).

Following reprogramming, RevitaCell™ Supplement was added to cells during each passaging step and resulted in higher cell attachment and more consistent passaging (data not shown).

### 3.3 Characterisation of iPSC

#### 3.3.1 Flow Cytometry

**FIGURE 3:**
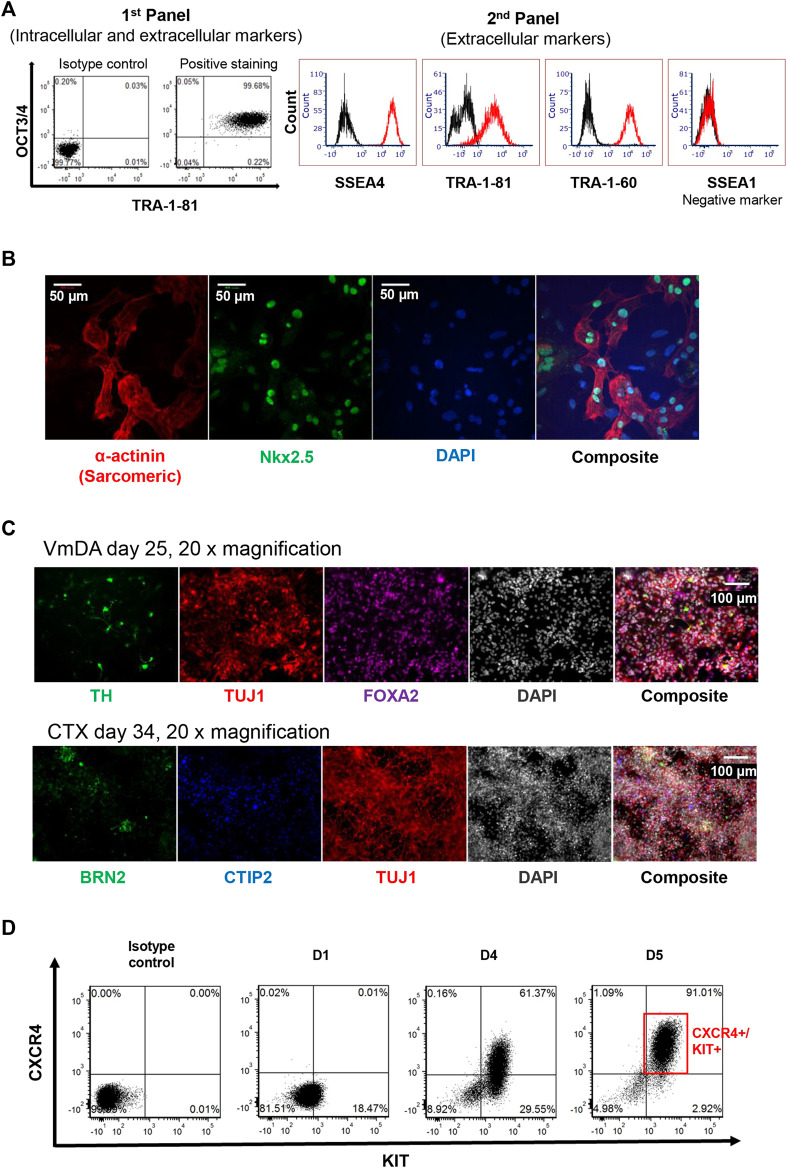
Assessment of iPSC pluripotency. **(A)** 2D plot of stemness markers used for assessing putative iPSC, including two panels used. **(B)** Immunofluorescence staining of cardiomyocytes derived from iPSC. Nkx2.5 shown in green, α-actinin (Sarcomeric) shown in red, DAP1 in blue. **(C)** Immunofluorescence staining of neuronal differentiation toward ventral midbrain dopamine (VmDA) neurons and cortical neurons from iPSC. TH and BRN2 shown in green, TUJ1 shown in red, FOXA2 shown in purple. CTIP2 shown in blue, DAPI shown in grayscale. **(D)** 2D plot showing 90% definitive endoderm cells could be obtained from iPSC cell line on day 5.

The pluripotency of iPSC was assessed by the presence of the widely accepted stemness markers TRA-1-81, TRA-1-60, OCT3/4, SSEA-4, along with the human stemness negative marker SSEA-1. During protocol optimisation, consistency of the expression of TRA-1-81^h^ on OCT3/4 positive cell population was observed by using panel 1 (Figure 3A). To simplify the screening process in-house, panel 2 of the combination of TRA-1-81 with other markers (SSEA-4 and TRA-1-60) was used instead of panel 1; iPSC colonies were more than 95% positive for these markers, and less than 2% positive for SSEA-1 (Figure 3A). These expression patterns were observed in more than 90% of screened colonies.

#### 3.3.2 Direct Differentiation of iPSC

To confirm that the iPSC lines created could be differentiated towards the mesoderm lineage, colonies were randomly selected and driven towards the cardiomyocyte lineage; beating cells were observed on day 10. These beating cells displayed the cardiomyocyte expression markers Nkx2.5 and α-actinin (Sarcomeric) using Immunofluorescence staining (Figure 3B).

To demonstrate that the iPSC line generated could be differentiated towards ectodermal lineage, cells were differentiated towards both ventral midbrain dopamine (VmDA) neurons and cortical neurons. The co-expression of OTX2/FOXA2 was observed on day 11 toward VmDA differentiation (data not shown), and tyrosine hydroxylase (TH), a rate-limiting enzyme in dopamine production, was detected among Class III β-tubulin (TUJ1) immature neurons on day 25 (Figure 3C). TUJ1 expressing neurons in cortical differentiations included cells expressing the deep layer cortical neurons marker CTIP2 and the upper layer cortical neurons marker BRN2 on day 34 (Figure 3C).

The same iPSC line was also differentiated towards the endoderm lineage. Flow cytometry analysis showed that around 91% definitive endoderm cells (CXCR4+/KIT^+^) could be obtained on day 5 of differentiation (Figure 3D).

These results demonstrate that iPSC lines created from cryopreserved cord blood under xeno-free, feeder-cell free conditions are pluripotent, with demonstrated differentiation towards all three germ layers.

### 3.4 Genome Integrity

iPSC lines from 5 individual colonies at early stage of passage were randomly selected for SNP array test. No aneuploidies were detected in 4 of the colonies. One colony had atypical genotyping profile in X chromosome, which could be segmental duplications or deletions along the X chromosome, at low level mosaicism (∼12–20%) or mosaic loss of heterozygosity.

### 3.5 Long-Term Maintenance of iPSCs Lines Under a cGMP-Compliant Cell Culturing System

**FIGURE 4:**
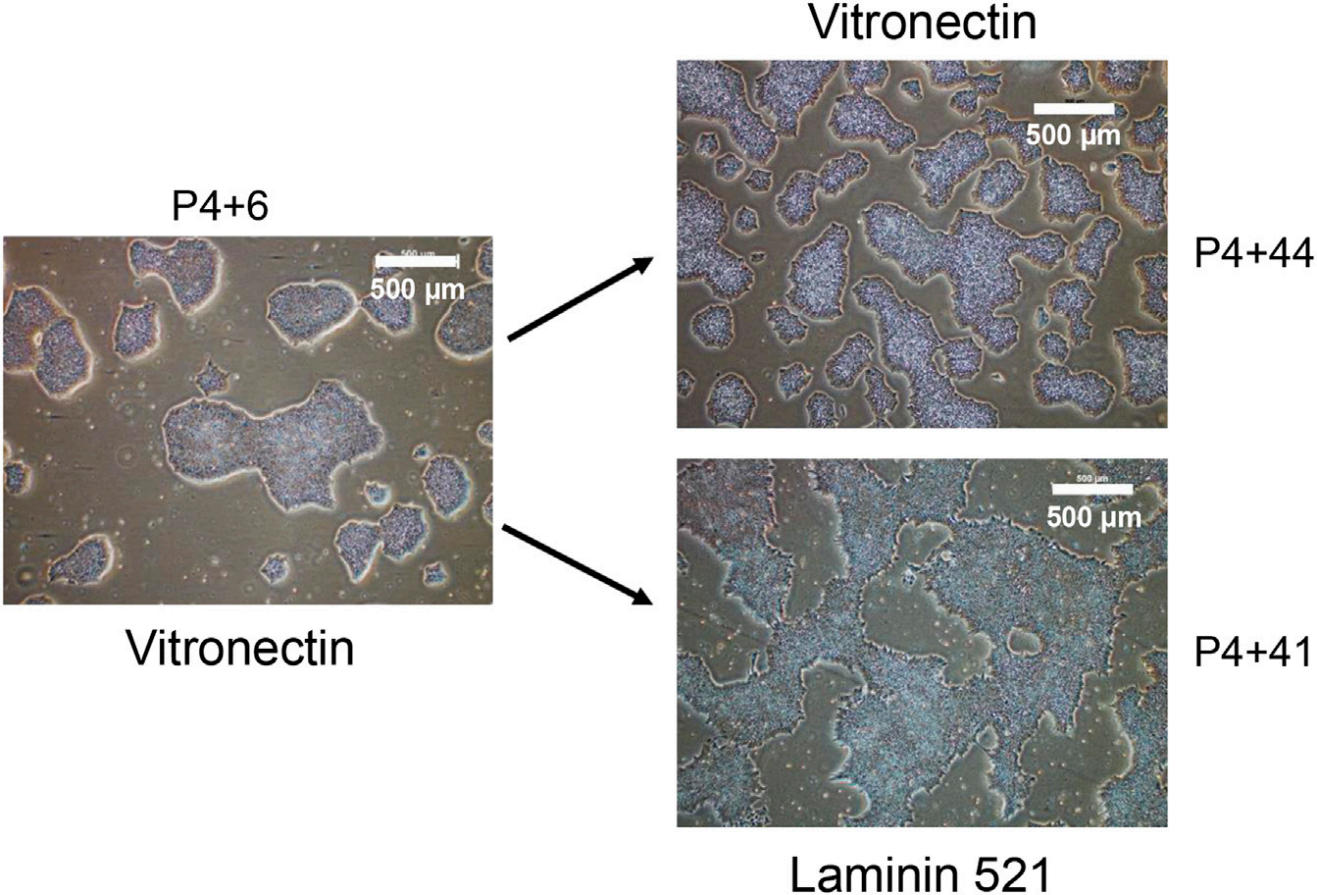
The morphology of iPSC colonies at early and late passage number. The generated iPSC cell line was maintained in parallel on Vitronectin and Laminin 521 coated flasks. P4 refers to the first 4 manual passages, with subsequent passages performed in bulk using Versene.

To determine whether iPSC lines created under xeno-free, feeder-layer free conditions could be maintained in *ex-vivo* culture long-term, and thereby assess stability of the lines, one of the iPSC lines that was characterised as described above for pluripotency and genomic integrity, and named MCRICBi001-A in accordance with hPSC nomenclature (https://hpscreg.eu/about/naming-tool), was continuously passaged on Vitronectin or Laminin 521 coated T25 flasks for more than 45 passages. A tight cell morphology was observed from cells maintained on Vitronectin and Laminin 521 through extensive cell passaging. However, cells grown on Vitronectin appeared tighter and smaller compared to the cells cultured on Laminin521, more closely physically resembling those of early passage cells (Figure 4).

The pluripotency of the cells maintained in culture to more than 45 passages was confirmed by flow cytometry. More than 98% of the cell population continued to demonstrate high expression of SSEA4, TRA-1-81 and TRA-1-60. Furthermore, these cells could be differentiated towards the mesoderm lineage, as indicated by beating cardiomyocytes (data not shown).

SNP array analysis was performed on cells at passage number 6, 23, and 37 maintained on Vitronectin or rhLaminin-521; no genomic abnormality (deletion and duplication) was detected (data not shown). These results indicate that genomic integrity and stability, at this level of assessment, could be maintained long-term under the xeno-free, feeder-layer free growth conditions.

The variation caused by different batches or lots of reagents was controlled in each step of iPSC generation and maintenance to ensure the robustness of this protocol.

No mycoplasma was detected in cell cultures.

We also conducted RT-PCR, as described in the manual for the CytoTune-iPS 2.0 Sendai Reprogramming Kit, to confirm the banked iPSC cell lines were Sendai virus vector-free. Some cell lines displayed a residue of vector backbone and Myc could be detected in most cases. The virus was eliminated by incubating the cells at 38.5°C for 4–5 days as per manufacturer’s recommendation (data not shown).

## 4 Discussion

The growing use of iPSCs in the regenerative medicine and cell therapy fields, has fed the demand for clinical grade iPSC lines suitable for translational use. In this study, we provide step-by-step cell culture instructions for creating GMP-compliant iPSC cell lines from a very small amount of banked CB. The success of any cell therapy application lies in the starting material. Banked CB from public CBB that meet compliance with their local Regulatory Authorities and, ideally, hold FACT accreditation, are an excellent source of starting material to create iPSCs for potential clinical use (Schwartz and Shaz, 2018). The BMDI CBB has a highly successful donor follow-up program, with CB donors re-consenting for their banked CB to be used to create iPSCs for potential therapeutic and commercial use (manuscript submitted). All aspects encompassing consent, ethics and a robust quality management framework are in place in accordance with worlds’ best practice (Steeg et al., 2021) to adopt the protocol described here for creation of GMP-grade CB-derived iPSC.

Whole CB consists of heterogeneous cell populations. Traditionally when blood cells are used for reprogramming, the red blood cells are removed and CD34^+^ cell isolation is performed to ensure that the high percentage of T cells harbouring variable T-cell receptors are not also reprogrammed. This procedure, however, would not be suitable for processing tiny volumes of cryopreserved CB due to the challenge of isolating the rare CD34^+^ progenitor cells for reprogramming (Baghbaderani et al., 2015). Instead, *ex-vivo* expansion was used to derive an erythroblast population as erythroid cells have been demonstrated as optimal target cells for reprogramming (Vlahos et al., 2019). After 7–9 days of *ex-vivo* expansion, erythroid cells were visually larger in size than other cell types and a gentle centrifugation speed was able to reduce the debris and contamination with cells that were smaller in size, which can affect reprogramming efficiency, while minimising erythroid cell loss. Undesirable reprogramming of T-cells was avoided using this *ex-vivo* expansion protocol. Flow cytometry confirmed that the T-cell population decreased to negligible numbers from day 7 onwards and the expanded cell population was comprised of more than 95% erythroid cells.

According to CytoTune 2.1 Sendai virus manufacturer’s instructions, one million cells should be used per reprogramming. Even with adoption of an optimised erythroid cell expansion protocol, it is unlikely that 10^6^ erythroid cells can be obtained from 200 µL CB on day 8–9 of *ex-vivo* expansion; in this protocol approximately only one fifth of the recommended starting cell number was reprogrammed. However, because of the higher reprogramming efficiency in cord blood MNC (personal observation) compared with peripheral blood MNC (Zhou et al., 2015), enough putative iPSC colonies could be obtained on the plates on day 19–21 post-transduction. We could easily observe 10 colonies with iPSC-like morphology per reprogramming.

More iPSC colonies were observed when the plates were incubated under hypoxic conditions compared with normoxic conditions, which was consistent with previous studies (Yoshida et al., 2009). We recommend maintaining reprogrammed cells in 5% O_2_ with 5% CO_2_ at 37°C from the time of plating the reprogrammed cells onto Vitronectin-coated dishes until stable iPSC clones are established and are undergoing EDTA-based passaging.

Flow cytometry was used as an easy way to characterise iPSC. TRA-1-80 bright cells were found to highly co-express OCT3/4. The percentage of TRA-1-80 bright cell population should be more than 90% for a good quality iPSC cell line and any cell lines that have more than 2% of SSEA1 positive cells should not be banked.

The application of RevitaCell™ Supplement (100 x) was extended from post-thaw recovery of cryopreserved cells to the passaging of hiPSC and provided a reproducible consistency in passaging. RevitaCell™ Supplement showed a higher consistency in improving survival of hiPSC in comparison with traditional ROCK inhibitors (i.e., Y-27632 and Thiazovivin) (personal communication in the lab), and has been shown to improve the of survival of hESC (Watanabe et al., 2007).

Both xeno-free matrices, rhLaminin 521, and Vitronectin, gave satisfactory results. However, differences in cell adhesion, growth rate and morphology were observed. While cells adhered better on rhLaminin 521, Vitronectin was selected instead of rhLaminin 521 for optimal growth and maintenance of iPSC cell lines. A recent publication reporting that cells with higher adhesion showed lower pluripotency (Yu et al., 2018), supported this choice. We also added the RevitaCell™ Supplement at half the recommended concentration to reduce the attachment selection pressure towards a differentiation direction during passaging. The long-term effects from RevitaCell are not known. However, RevitaCell is very similar to Y-27632 ROCK inhibitor, which is routinely used as a supplement by CiRA for growth of cells for feeder-free culture. In our laboratory, we assessed the genomic integrity using SNP array for one of the described cell lines at a very late passage and did not observe any genomic abnormalities.

Optimisation of cell confluency and passage frequency during maintenance was found to be critical. When iPSC were grown to over 70% confluence on Vitronectin, or iPSC were cultured for more than 4 days, some differentiating cells could be observed at the later passages (data not shown). Thus, iPSC over-confluency or culturing should be avoided in this fully defined culture system.

It was important to perform the genomic integrity assessment (SNP array-based) routinely. In this study, one clone was found to harbour genomic abnormality among 5 samples tested. From personal communication with the MCRI iPSC core lab, the average rate of reprogrammed iPSC clones harbouring mutations is around 10% (based on hundreds of clones tested). This highlights the importance of including genomic integrity assessment as part of standard quality control tests. Karyotype testing was not performed in this proof-of-concept study, however would be required for qualification of clinical-grade cell lines.

The clinical version (CTS) of CytoTune™ 2.1 Sendai virus was recently released and is the first off-the-shelf reprogramming kit. In this study, for financial considerations the research version of this kit was used. Nonetheless, a high reprogramming efficiency was observed with 200 µL of CB buffy and an ample number of iPSC colonies obtained per reprogramming.

The impact of long-term culturing under xeno-free, feeder-free conditions was investigated. The robust iPSC culturing and passaging system established in this study is suitable for long term culture.

Throughout the course of protocol development and validation, some vital observations were made and are provided here as tips to ensuring a successful outcome. These are:

(i) Always bring medium and buffers to room temperature prior to use.
(ii) If possible, conduct iPSC reprogramming and maintenance in low O_2_ (5%) culture conditions.
(iii) Never over-grow iPSC cultures: passage the cells when the cells reach 60–70% confluency or on day 3 or 4. Do not culture the cells for more than 4 days in the same flask.
(iv) Dry EDTA passaging of cells should take place for 3 min at 37°C (1 min after addition of dissociation buffer +2 min after removal of dissociation buffer). There is no need to prolong the incubation time as stickier cells tend to differentiate and are better left behind.
(v) Add 1:200 dilution RevitaCell™ Supplement into medium during passaging, then remove it within 24 h

The protocols developed and described herein can be easily adopted into a cGMP-compliant production pipeline for potential clinical translation application. The starting material is CB from a TGA-licensed and FACT-accredited facility. Each element used in this system is a commercially available product, chemically defined and from the reprogramming step onwards are from the CTS suite of products available from ThermoFisher, which are GMP-compliant with master files (Table 1). The catalogue numbers of CTS-grade products are shown in Table 1. We have described a protocol that is accessible to all in the field. Adoption of this protocol, with heed given to tips provided, allows efficient and robust creation of clinical-grade iPSC cell lines from small volumes of cryopreserved CB.

## Data Availability

The data presented in this study are deposited in the European Variation Archive repository https://www.ebi.ac.uk/, accession number PRJEB51091. The raw data supporting the conclusions of this article will be made available by the authors, without undue reservation.
