# Supplementary figures and images for: Creation of GMP-Compliant iPSCs From Banked Umbilical Cord Blood
Source: Front Cell Dev Biol. 2022 Mar 16;10:835321. doi: 10.3389/fcell.2022.835321 (PMC8967326; doi:10.3389/fcell.2022.835321)

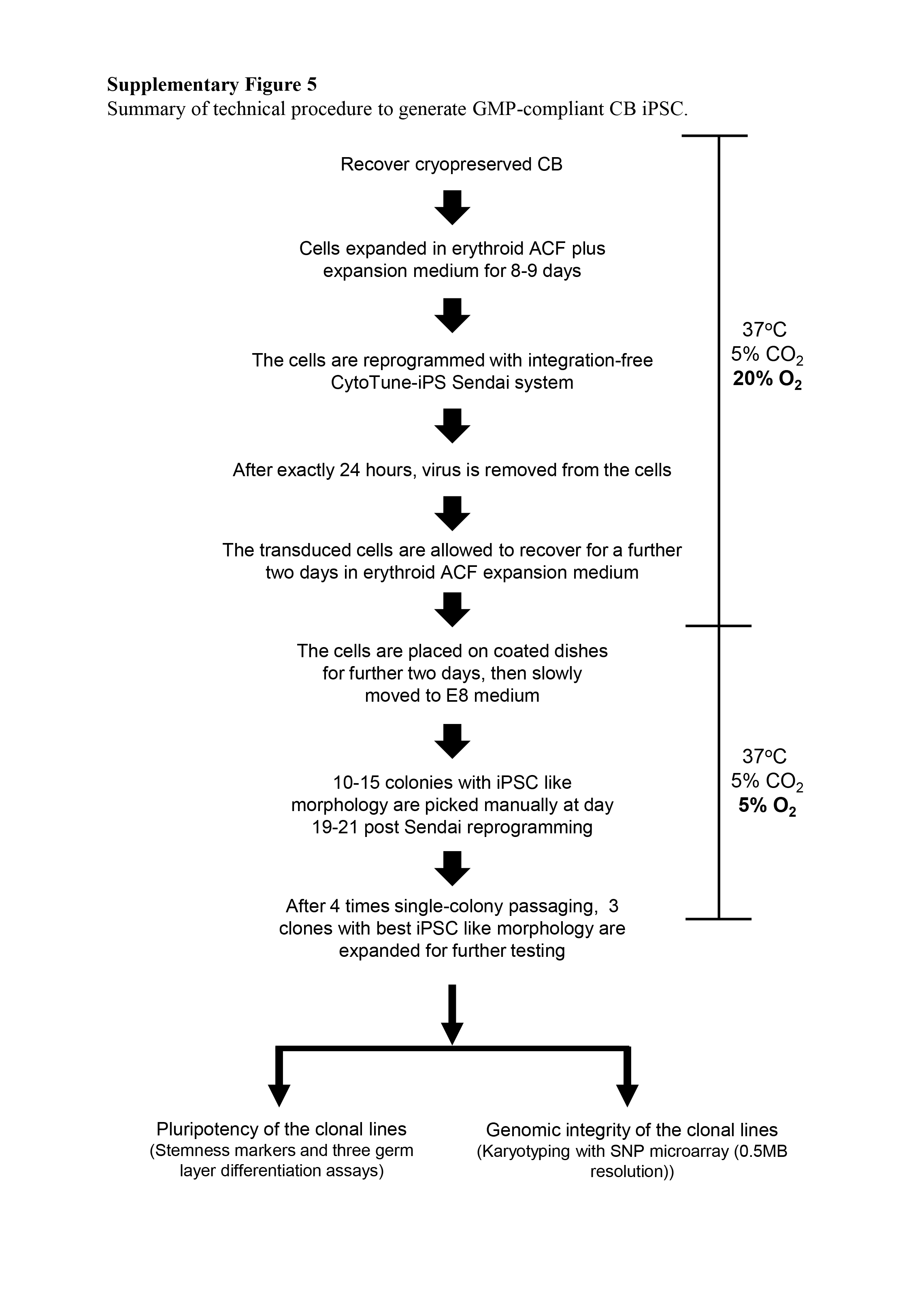

Supplement: Supplementary file 1 [file Image5.jpg]

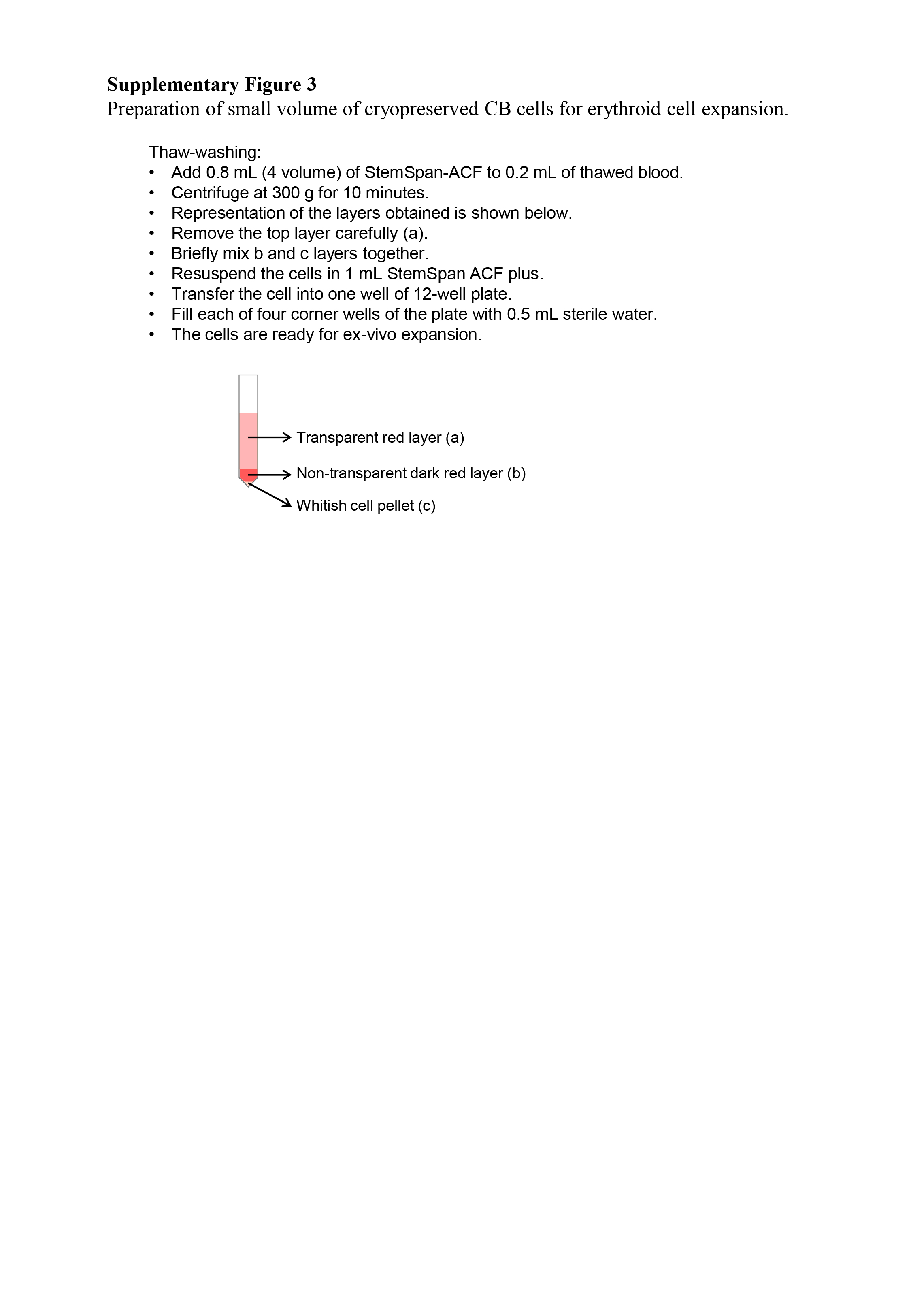

Supplement: Supplementary file 2 [file Image3.jpg]

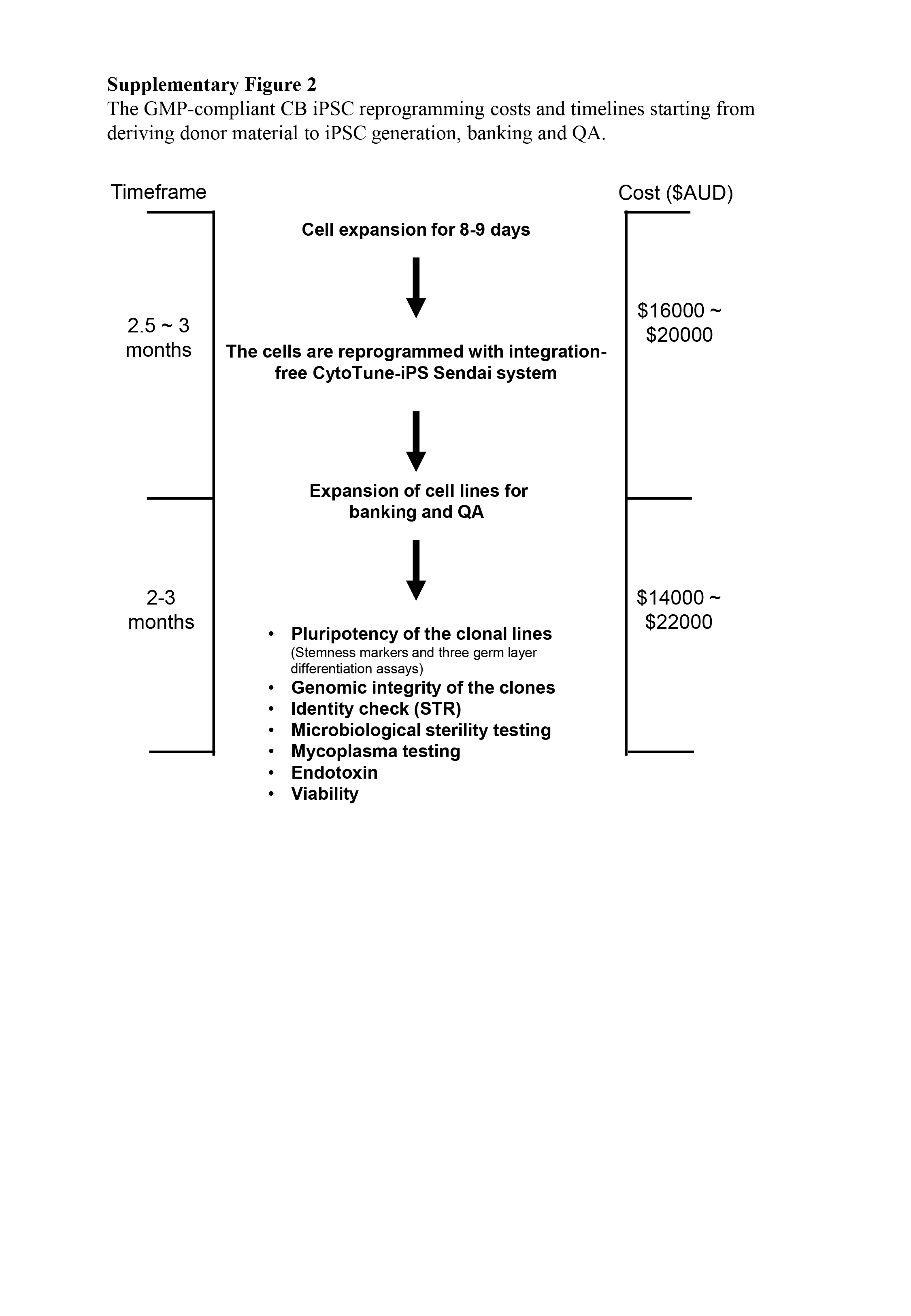

Supplement: Supplementary file 3 [file Image2.jpg]

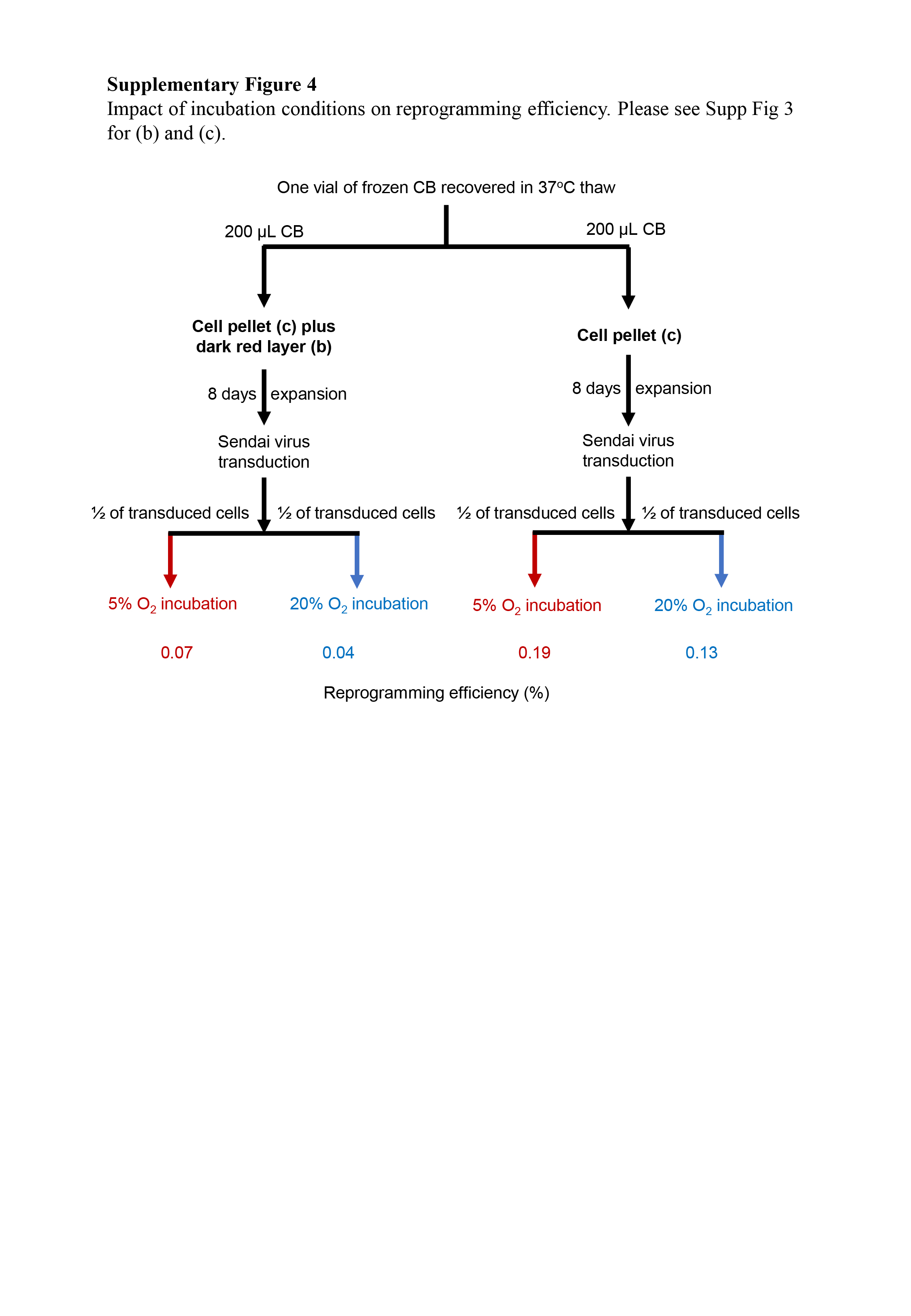

Supplement: Supplementary file 5 [file Image4.jpg]

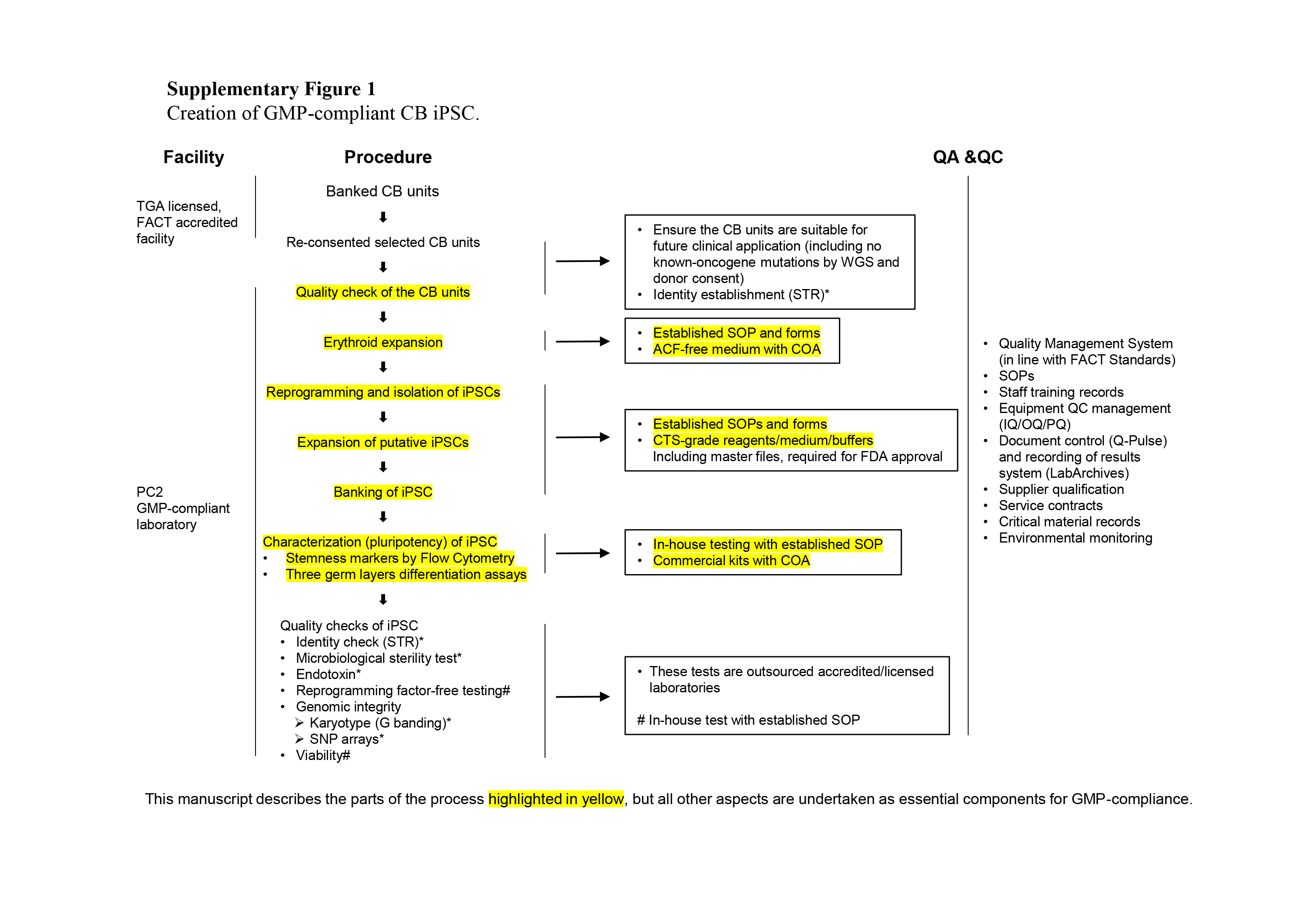

Supplement: Supplementary file 6 [file Image1.jpg]
