# Supplementary material for: Creation of GMP-Compliant iPSCs From Banked Umbilical Cord Blood
Source: Front Cell Dev Biol. 2022 Mar 16;10:835321. doi: 10.3389/fcell.2022.835321 (PMC8967326; doi:10.3389/fcell.2022.835321)
Supplement: Supplementary file 4 [file DataSheet2.docx]

Supplementary Material

# Supplementary methods

**Details of In vitro directed differentiation assay**

To examine the pluripotency of the putative iPSC lines, the reprogrammed cells were differentiated towards three germ layers: neural stem cell (NSC) (ectoderm), cardiomyocytes (mesoderm) and definitive endoderm as described below.

For neuronal differentiation, hPSCs were seeded into Laminin-521-coated (5 μg/mL) plates at 0.375 × 10^6^/cm^2^ in mTeSR1 medium with 10 μM Rock inhibitor Y-27632 (Tocris Bioscience). Neural induction was commenced 24 hours later by dual SMAD inhibition.

The ventral midbrain (VM) differentiation procedure has been described in detail (Gantner et al., 2020). Briefly VM patterning was simultaneously achieved by supplementation of the media with sonic hedgehog (200 ng/mL, 1-7DIV; R&D Systems), purmorphamine (2 mM, 1–7DIV; Stemgent) and CHIR99021 (3 mM, 3–13DIV; Miltenyi Biotech). From 11DIV, cultures were matured in the presence of brain-derived neurotrophic factor (BDNF, 20 ng/ml, R&D Systems), glial cell line-derived neurotrophic factor (GDNF, 20ng/ml, R&D Systems), recombinant human transforming growth factor type b3 (TGFb3, 1ng/ml, Peprotech), ascorbic acid (200nM, SigmaAldrich), dibutyryl cAMP (0.05 mM, Tocris) and DAPT (10 μM, Sigma-Aldrich). Immunocytochemistry was conducted on day 11 and day 25.

Generation of cortical neurons was as described previously in detail (Gantner et al., 2021) based on neural induction via dual SMAD inhibition using 100 nM LDN193189 (Stemgent) and 10 μM SB431542 (R&D Systems) for 11 days. On D11, progenitors were passaged and reseeded 1:2.5 in cortical growth medium consisting of 1:1 DMEM/F12 and Neurobasal with 0.5× B27, 0.5× N2, 0.5× ITSA, 1× GlutaMAX, 0.5 × penicillin/streptomycin, and 50 μM 2-mercaptoethanol (Life Technologies). On D11, progenitors were passaged and reseeded 1:2.5. Cells were fixed for immunocytochemistry on day 34.

For immunocytochemistry, cultures were fixed with 4% PFA at day 11 and day 25 for VM differentiation and day 34 for cortical induction. Immunohistochemistry was performed in accordance to previously described methods (Somaa et al., 2017). Primary antibodies and dilutions were as follows: DAPI (1:5000; Sigma-Aldrich), rabbit anti-OTX2 (1:4000; Millipore), goat anti-FOXA2 (1:200; Santa Cruz), sheep anti-TH (1:800, Pelfreeze), mouse anti-TUJ1 (Promega), Rat anti-CTIP2 (Abcam), Goat anti-BRN2 (Santa Cruz). Secondary antibodies for direct detection were used at a dilution of 1:200: DyLight 488-, 549-, or 649-conjugated donkey anti-mouse, anti-rabbit, anti-goat, anti-rat or anti-sheep (Jackson Immuno Research Laboratories). The images were acquired using Zeiss LSM780 confocal microscope.

Directed differentiation of cardiomyocytes was achieved by following a previously published method (Lian et al., 2013). A similar result was subsequently obtained using the commercially available kit PSC Cardiomyocyte Differentiation Kit (Gibco A29212-01).

Reprogrammed lines were seeded at 2.5×10^5^ cells per well on 24-well Geltrex™ (Gibco A1413201) coated plates in Essential 8 medium three days prior and medium was refreshed daily. On day 0 of differentiation, Essential 8 medium was replaced with RPMI plus B-27 without insulin (Life Technologies 0050129SA) plus GSK3 inhibitor CHIR99021 (Stemgent, 12 μM) for 24 h. On day 1, CHIR99021 was removed and replaced with RPMI plus B-27 without insulin until day 3. On day 3, medium was replaced with RPMI plus B-27 without insulin plus Wnt inhibitor 2 (IWP2, Stemgent, 5 μM) and the cells were incubated for 48 h until day 5. On day 5, IWP2 was removed and replaced with RPMI plus B-27 without insulin until day 7. On day 7, medium was replaced with RPMI plus B-27 and subsequently the cells were maintained in RPMI B-27 and media refreshed every 3 days. Beating cells could be observed on day 10-14. Differentiated cells were stained with cardiomyocyte markers, rabbit anti-NKX2.5 (1:250; Abcam ab35842) and mouse anti-α-actinin (Sarcomeric) (1:250; Sigma-Aldrich A7811). Secondary antibodies for detection were used at a dilution of 1:500 for Goat anti-Rabbit IgG Alexa-647 (ThermoFisher A-21245) and 1:500 for Goat anti-Mouse IgG Alexa-568 (ThermoFisher A-11031). The images were acquired using Dragonfly Spinning Disk Confocal Microscope.

Definitive endoderm was induced according to a previously published method (Loh et al., 2014). At approximately 20% confluency the iPSC cells were plated on Geltrex-coated 6-well plate in E8 medium at 37oC, 5% CO2 overnight. The next day (day 0), E8 medium was replaced with 2 mL of STEMdiff ™ APEL ™ Medium (StemCell Technologies 05210) containing 2 μM CHiR99021 (Sigma-Aldrich 252917-06-9), 100 μg/mL Activin A (R&D 338-AC-010), 50 μM PI-103 kinase inhibitor (Cayman Chemical 371935-74-9). The media was then changed daily with STEMdiff ™ APEL ™ Medium containing 1 µM DMH-1 (Sigma-Aldrich 1206711-16-1) and 100 μg/mL Activin until day 5.

The differentiated cells were analysed using Flow Cytometry. The cells were stained with 50 µL of antibodies cocktail mix containing BV421-conjugated CXCR4 (1:25; B&D 562448) and PE-Cy7 conjugated KIT (CD117) (1:50; B&D 339195).

**References**

Gantner, C.W., De Luzy, I.R., Kauhausen, J.A., Moriarty, N., Niclis, J.C., Bye, C.R., et al. (2020). Viral Delivery of GDNF Promotes Functional Integration of Human Stem Cell Grafts in Parkinson's Disease. *Cell Stem Cell* 26**,** 511-526.e515.

Gantner, C.W., Hunt, C.P.J., Niclis, J.C., Penna, V., Mcdougall, S.J., Thompson, L.H., et al. (2021). FGF-MAPK signaling regulates human deep-layer corticogenesis. *Stem cell reports* 16**,** 1262-1275.

Lian, X., Zhang, J., Azarin, S.M., Zhu, K., Hazeltine, L.B., Bao, X., et al. (2013). Directed cardiomyocyte differentiation from human pluripotent stem cells by modulating Wnt/beta-catenin signaling under fully defined conditions. *Nat Protoc* 8**,** 162-175.

Loh, K.M., Ang, L.T., Zhang, J., Kumar, V., Ang, J., Auyeong, J.Q., et al. (2014). Efficient endoderm induction from human pluripotent stem cells by logically directing signals controlling lineage bifurcations. *Cell Stem Cell* 14**,** 237-252.

Somaa, F.A., Wang, T.Y., Niclis, J.C., Bruggeman, K.F., Kauhausen, J.A., Guo, H., et al. (2017). Peptide-Based Scaffolds Support Human Cortical Progenitor Graft Integration to Reduce Atrophy and Promote Functional Repair in a Model of Stroke. *Cell Rep* 20**,** 1964-1977.
